# Antibacterial and Cytotoxic Silica-Polycaprolactone-Chlorogenic Acid Hybrids by Sol–Gel Route

**DOI:** 10.3390/molecules28083486

**Published:** 2023-04-15

**Authors:** Michelina Catauro, Antonio D’Angelo, Veronica Viola, Giovanna Cimmino, Severina Pacifico

**Affiliations:** 1 Department of Engineering, University of Campania “Luigi Vanvitelli”, Via Roma 29, I-81031 Aversa, Italy; 2 Department of Environmental, Biological, and Pharmaceutical Sciences and Technologies, University of Campania “Luigi Vanvitelli”, Via Vivaldi 43, I-81100 Caserta, Italy

**Keywords:** sol–gel, chlorogenic acid, FTIR spectroscopy, bioactivity, biocompatibility

## Abstract

Organic–inorganic hybrid materials were synthesized by a sol–gel route, using silicon alkoxide together with low molecular weight polycaprolactone and caffetannic acid. The synthesized hybrids were characterized by scanning Fourier-transform infrared (FTIR) spectroscopy, and their surface morphology was acquired by scanning electron microscopy (SEM) analysis. The hybrids were investigated for their antiradical capacity using the DPPH and ABTS tests, while the Kirby–Bauer test was used to evaluate their effects on the growth of *Escherichia coli* and *Enterococcus faecalis*. Furthermore, a biologically active hydroxyapatite layer has been observed to form on the surface of intelligently synthesized materials. The MTT direct test showed that the hybrid materials are biocompatible with NIH-3T3 fibroblast cells, while they were cytotoxic towards colon, prostate, and brain tumor cell lines. These results shed new light on the suitability of the synthesized hybrids in the medical field, thus affording knowledge on the features of the bioactive silica–polycaprolactone–chlorogenic acid hybrids.

## 1. Introduction

Low molecular weight natural substances, biosynthetically derived from the secondary metabolism of plants, are receiving particular attention in the chemistry of materials, since the incorporation/trapping of these compounds can lead to an improvement of their physicochemical properties and bioactivity for a wide range of applications [1,2,3]. The possibility of designing materials through the hybridization of organic and inorganic components has been attentively studied, providing multifunctional materials [4]. These materials, also called hybrids, being a combination of at least two components that integrate at the molecular level, can be exploited in different ways, through the synthetic approach adopted and the control of the parameters of influence [5,6,7,8].

One of the approaches used to synthetize hybrid materials is the sol–gel technique. This is a versatile technique that involves the conversion of a liquid sol (a colloidal suspension of inorganic particles) into a solid gel, which can then be further processed to obtain the desired material [9,10,11]. It is generally accepted that all the reaction mechanisms that govern sol–gel chemistry are based on second-order nucleophilic substitutions (S_N_2) [12] and that the whole process typically involves four steps: (i) sol preparation; (ii) gel formation; (iii) aging; and (iv) drying. During the first step, a precursor solution, prepared by dissolving metal alkoxides or metal salts in a suitable solvent, is hydrolyzed to form a sol, which is a colloidal suspension of inorganic particles. As the hydrolysis reactions go on, the second step starts. This is mainly based on polycondensation reactions, which involve the formation of a three-dimensional network of interconnected particles once it has reached the gelation point. The gelation process can be triggered by various factors, such as changes in temperature, pH, or the addition of a crosslinking agent. After the gelation has occurred, the gel is then aged for some time to promote further crosslinking and consolidation of the network. This step can take several hours or even days, depending on the specific materials and conditions used. The last step involves drying treatments of the gel to remove the solvent and obtain the final glassy-ceramic material. This can be done by various methods, such as evaporation, supercritical drying, or freeze-drying. By carefully controlling the sol–gel parameters, such as precursor composition, solvent, pH, and temperature, it is possible to tailor the properties of the resulting materials, such as porosity, surface area, mechanical strength, and optical properties [13,14,15,16]. In particular, the low reaction temperatures also make available, in the presence of a properly enriched organic polymeric component and bioactive molecules, the incorporation of organic fractions into inorganic materials [17].

In this framework, several investigations have been carried out to evaluate the possibility of incorporating polymeric compounds and bioactive molecules with a polyphenolic backbone in a polymer–silica hybrid network, constituting bioactive glasses characterized by their ability to form a direct chemical bond with bone at an implant site through the mineralization of a biomimetic apatite layer [18,19,20]. Of particular interest is a hybrid material containing polycaprolactone (PCL). Although PCL could have some undesirable characteristics, such as low reactivity, hydrophobicity, and slow degradation rate, its suitability depends on the specific application requirements. Indeed, PCL in biohybrid materials has been used mainly for its biodegradability, biocompatibility, mechanical properties, and chemical stability [21,22,23,24,25,26]. For example, PCL nanofibers loaded with caffeic acid and epigallocatechin gallate were observed to exert an anti-tumor activity, while the incorporation of curcumin favored an improvement of osteoblastic cell functions [22]. The formulation of quercetin-loaded microspheres using low molecular weight PCL have been used in the management of inflammation and other associated symptoms and in slowing the progression of cartilage degradation [26]. A hybrid matrix of silica and poly(ɛ-caprolactone) by a sol–gel route equally trapped the antioxidant agent quercetin, constituting materials in which PCL, due to its intrinsic plasticizing capacity, imparted elasticity, thus reducing the brittleness of sol–gel materials [27,28]. Antimicrobial properties towards *Escherichia coli* and *Pseudomonas aeruginosa* of the bioactive silica–PCL hybrids were also observed following the embedding of chlorogenic acid [29]. This latter compound, also known as 5-*O*-caffeoyl quinic acid, is a natural constituent of dietary plant sources, mainly coffee, coffee beans [30], and spices or sweetener plants [31] broadly used in the food sector [32,33]. The antimicrobial properties are not the only ones that make this compound highly functional. In fact, other studies showed its interest as a nutraceutical and as an agent for the preservation and conservation of food products [34,35]. Indeed, as an anti-lipoperoxidant agent, it is useful in delaying rancidity, and by exerting an antioxidant and prebiotic action, it lends itself to being a good candidate for the formulation of food supplements and functional foods [36]. The interest in this compound goes much farther, since chlorogenic acid (CGA) exerts positive effects at several levels on the gastrointestinal, cardiovascular, and liver systems, and has been shown to be a scavenger of free radicals and a stimulator of the central nervous system [37]. The functionality of chlorogenic acid has also aroused interest in the context of active food packaging and biomaterials, so much so that hybrids based on chlorogenic acid have been suitably synthesized [38,39,40]. While chlorogenic acid–silica hybrids [41] and chlorogenic acid(20 wt%)–silica–PEG/PCL materials have already been synthetized [42], no study has been conducted before on low percentages of the chlorogenic acid–silica–PCL systems. The aim of this study is, therefore, to synthetize, using the sol–gel method, hybrid materials composed of silica as an inorganic component, polycaprolactone (from 0 to 24 wt%) as an organic polymer, and chlorogenic acid as a bioactive phenolic compound at different percentages (from 0 to 15 wt%). The hybrids were named according to the following label SCxPy, where S = SiO_2_, C = CGA, P = PCL, and x and y are the weight percentages of the chlorogenic acid and the PCL, respectively. The synthetized materials were chemically characterized by Fourier-transform infrared spectroscopy. The bioactivity of the hybrids was also analyzed by FTIR spectroscopy and scanning electron microscopy coupled with energy dispersive spectroscopy (EDS). The hybrids underwent antiradical and antimicrobial assessment (against *Escherichia coli* and *Enterococcus faecalis*, known as bacteria that cause nosocomial infections [43,44,45]), while their biocompatibility was determined by the direct contact MTT assay on NIH-3T3, Caco-2, DU-145, and SH-SY5Y cells.

## 2. Results and Discussion

### 2.1. Characterization of the Synthesized Materials by FTIR

**Figure 1:**
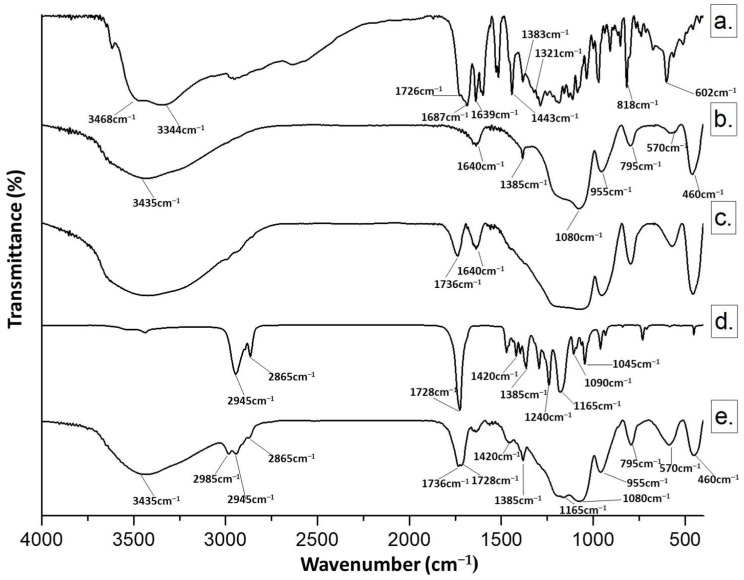
Representative FTIR spectra of (**a**) CGA (C); (**b**) pure SiO_2_ (S); (**c**) SC15; (**d**) pure PCL (P); (**e**) SC15P24. The number indicates the wt% of CGA and/or PCL in hybrids.

The hybrid materials were investigated using FTIR spectroscopy to obtain information on the chemical bonds involved in the hybrids. Representative FTIR spectra of (a) CGA, (b) pure SiO_2_, (c) SC15 hybrid, (d) pure PCL, and (e) SC15P24 are reported in Figure 1.

When chlorogenic acid was added to the silica matrix (Curve c), a red shift (+10 cm^−1^) was observed at the level of the carbonyl-stretching frequencies, according to the establishment of H-bonds with silica matrix [46], while the band at 1640 cm^−1^ was attributable to chlorogenic acid aromatic ring vibrations, and bands in the fingerprint region appeared to be superimposable on those in the silica matrix spectra [47,48,49,50].

Polycaprolactone incorporation (Curve e) gave rise to a spectrum with its characteristic peaks at 2945 cm^−1^ and 2865 cm^−1^, due to CH_2_ and CH_3_ stretching vibrations, and a weak signal at 1385 cm^−1^, attributable to their bending mode [29,51]. The polymer CO stretching vibration appeared unmodified at 1728 cm^−1^, whereas silica bands at 1080 and 460 cm^−1^ due to stretching and bending of Si–O–Si, as well as the band at 955 cm^−1^ related to Si–OH stretching, were also detectable [52,53,54,55], along with bands at 1385 and 570 cm^−1^ as a result of residual four-membered siloxane rings in the silica network [56,57].

**Figure 2:**
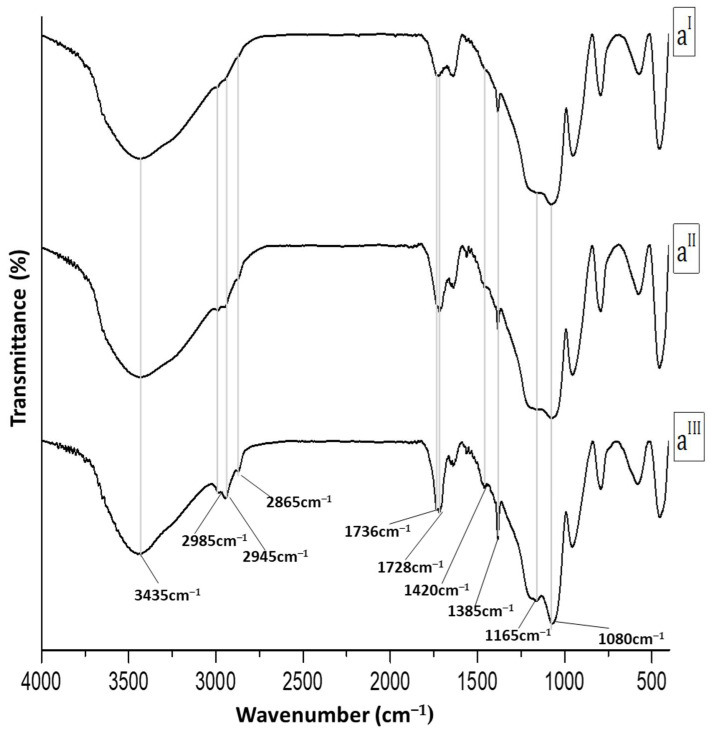

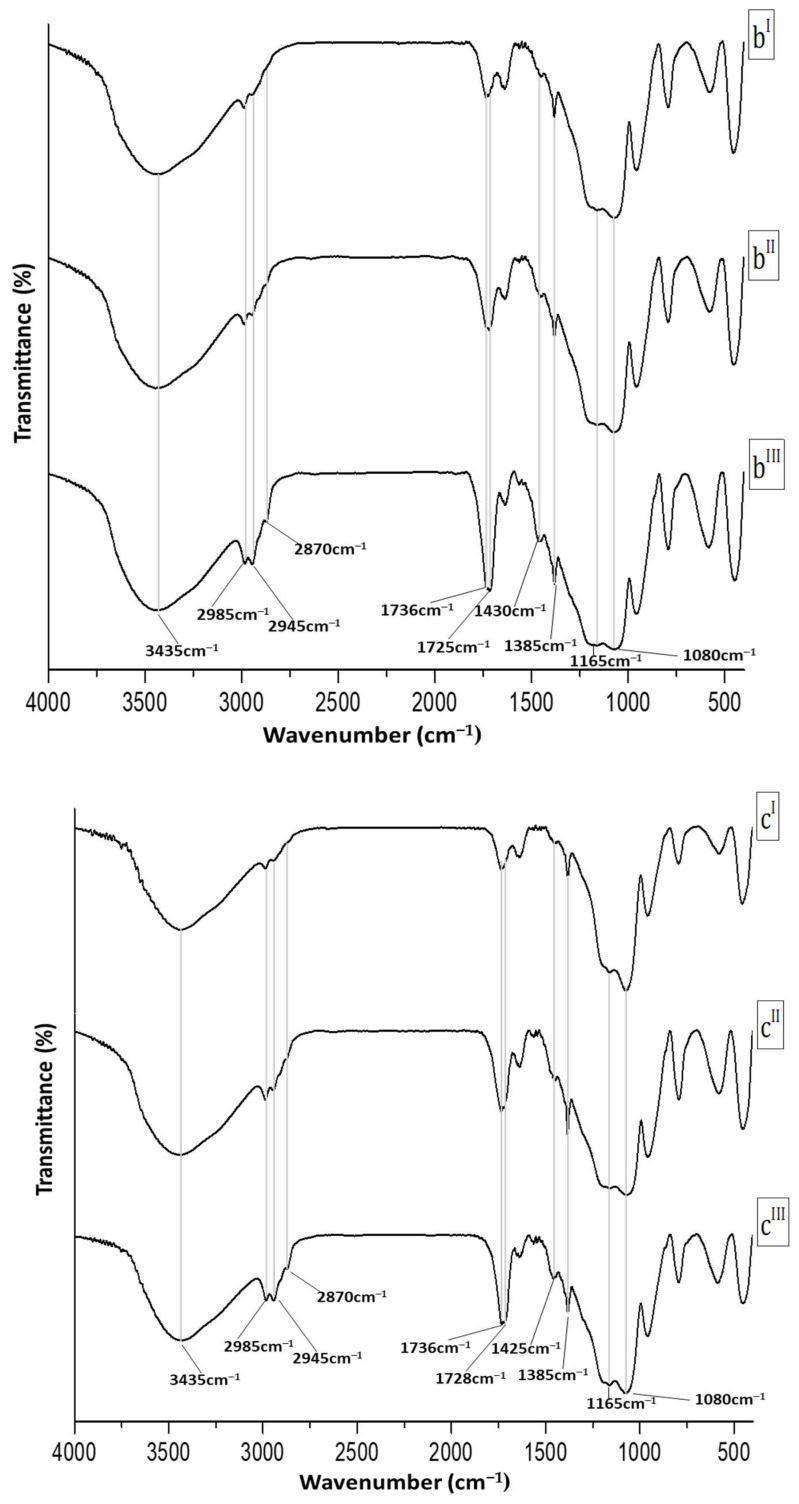
FTIR spectra of (**a^I^**) SC5P6; (**a^II^**) SC5P12; (**a^III^**) SC5P24; (**b^I^**) SC10P6; (**b^II^**) SC10P12; (**b^III^**) SC10P24; (**c^I^**) SC15P6; (**c^II^**) SC15P12; (**c^III^**) SC15P24.

The increase in polycaprolactone content from 6 wt% to 24 wt% provided spectra with peaks of correlatively higher intensity (Figure 2). Indeed, it can be noted that the polymer content increase, regardless of the percentage of chlorogenic acid, provides spectra with distinguishable bands of methylene functions, especially at the highest polymer percentage. Furthermore, the ratio of the band intensities at 1736, 1728, and 1640 cm^−1^ appeared to be closely related to the percentage of individual constituents in the hybrid.

### 2.2. Bioactivity Test

After soaking hybrid materials in SBF for three weeks, bioactivity was evaluated by detecting hydroxyapatite [Ca_10_(PO_4_)_6_(OH)_2_] nucleation on sample surfaces. This could be due to the ability of Si–OH groups on the surface to attract Ca^2+^ ions in simulated body fluid. When Ca^2+^ ions combine with phosphate ions, amorphous calcium phosphate is formed and spontaneously transforms into hydroxyapatite [58,59].

**Figure 3:**
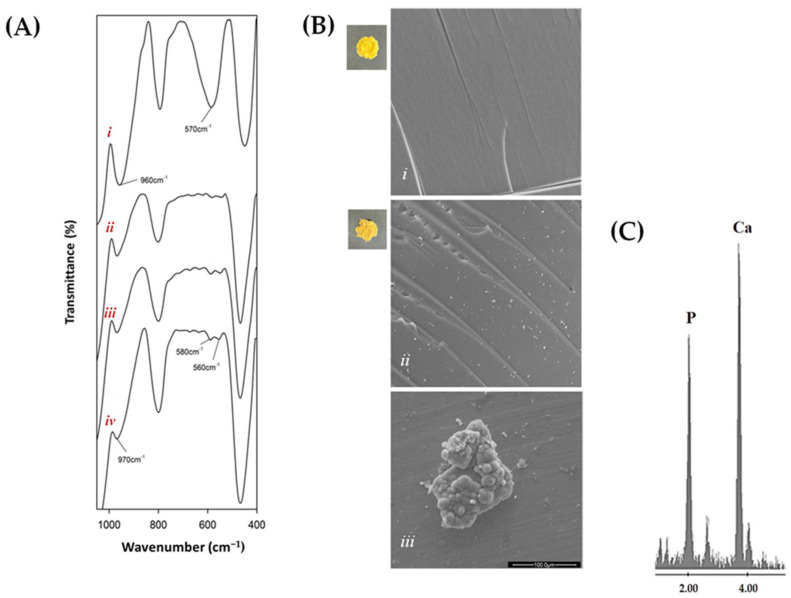
(**A**) FTIR spectra of (***i***) SiO_2_; (***ii***) SC10P6; (***iii***) SC10P12; (***iv***) SC10P24; (**B**) representative SEM micrographs of (***i***) SC10, (***ii***) SC10P12, and (***iii***) SC10P12, after the three-week soaking in SBF. (**C**) EDS spectrum of the SC10P12 hybrid following the three-week exposure to SBF.

FTIR spectra (Figure 3A) of samples soaked in SBF solution showed that the band at 570 cm^−1^ (Panel *i*; silica spectrum) gave up bands at 575 cm^−1^ and 560 cm^−1^ (Panels *ii*, *iii*, and *iv*; SC10Py hybrids), attributable to phosphate ion vibrations, which were followed by hydroxyapatite precipitation [60,61]. Furthermore, the red shift of the Si–OH band suggested the interaction of the hydroxyapatite layer with the –OH groups of the silica matrix [62] (Figure 3A).

Representative images of the surface morphology of the hybrids, acquired by SEM analysis before and after three weeks of exposure to SBF, are shown in Figure 3B. In particular, Panels *i* (showing SC10 hybrid) and *ii* (showing SC10P12 hybrid) highlighted that no significant differences were observed between the different materials, as the structure appears uniform and all systems are homogeneous with no phase separation even at high magnifications, in line with the incorporation of the phenolic compound into the silica–polycaprolactone network (Figure 3B(*ii*)). The nucleation of hydroxyapatite into the SBF allowed its growth to occur on the surface of hybrids (Figure 3B(*iii*)). This was further confirmed by EDS analysis (Figure 3C). Indeed, the nucleated globular-shaped hydroxyapatite on the SC10P12 hybrid consists of an elemental composition of Ca and P, with an atomic ratio equal to 1.67.

### 2.3. SCxPy Materials Showed Chlorogenic-Acid-Content-Dependent Antiradical Efficacy

**Figure 4:**
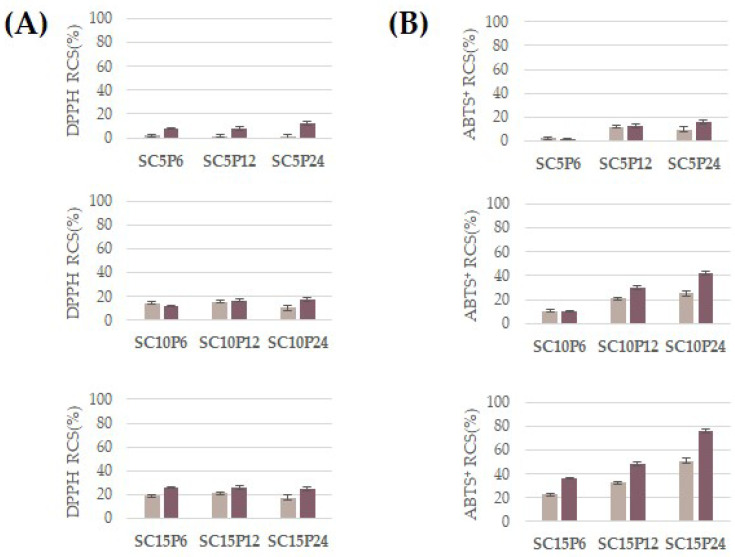
Radical Scavenging Capacity (RSC, %) of hybrids directly exposed to DPPH^●^ (**A**) and ABTS^●+^ (**B**) at 1.00 mg dose (●) and 2.00 mg dose (●). Values are the mean ± SD of measurements carried out on three samples analyzed three times.

The interaction between the various components leads to the creation of hybrids with weak antiradical effects, especially when they contain a low percentage of chlorogenic acid (Figure 4). Indeed, evaluating the antioxidant capability based on the activity of relative SCxPy hybrids, it was found that the addition of chlorogenic acid into SCxPy hybrids resulted in improved free radical inhibition. The antiradical efficacy increased as the chlorogenic acid content increased. In particular, the ABTS radical cation was mostly scavenged after reacting with the SC15P24 sample, underlining that polycaprolactone at the highest dose also contributes to the activity exercise. The effect was mainly observable when hybrid powders were tested at a 2.00 mg dose.

### 2.4. Antibacterial Properties of Hybrid Materials

**Figure 5:**
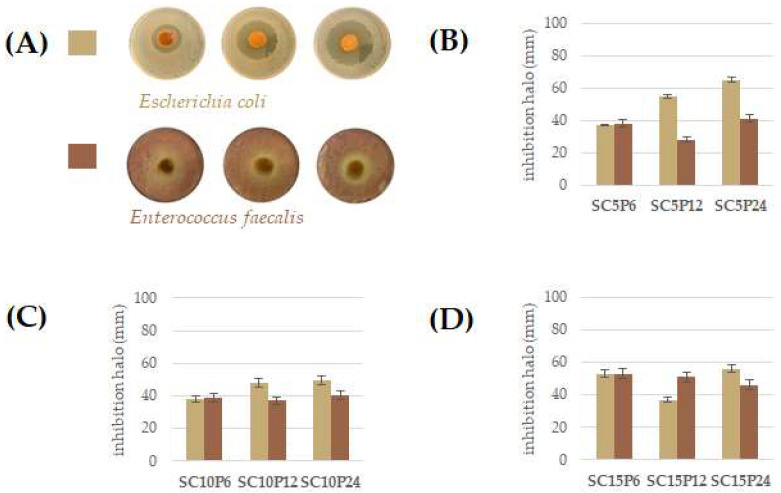
(**A**) Representative images of detected zone of inhibition around bacterial colonies of (●) *Escherichia coli*, and (●) *Enterococcus faecalis*. Inhibition halo (mm) of *Escherichia coli* and *Enterococcus faecalis* with SCP hybrids at chlorogenic acid percentage equal to 5wt% (**B**), 10 wt% (**C**), and 15 wt% (**D)**. Values are calculated as mean ± SD of three independent measurements.

The SCxPy hybrids’ inhibitory effects on microbial growth is reported in Figure 5A–D. SCxPy hybrids were able to inhibit the growth of *Escherichia coli*, a Gram-negative, rod-shaped bacterium [63,64]. In particular, the growth of this facultative aerobe, which, bearing a sensor for oxygen presence, can activate or repress the required metabolic enzymes, appeared to be mainly inhibited by SC5Py hybrids at the lowest content in chlorogenic acid (Figure 5B). The effect was found to increase when increasing the polymer content.

*Enterococcus faecalis*, a Gram-positive bacterium, whose cell wall was constituted by the three components of peptidoglycan, teichoic acid, and polysaccharide [65], was inhibited mostly by SC15Py hybrids, highlighting that chlorogenic acid could be the main actor of the detected effect. In this context, chlorogenic acid was proven to exert antimicrobial activity by changing the permeability of microbial cell walls [66], and its bactericidal activity was against both Gram-positive and -negative bacteria [67,68].

### 2.5. Cytotoxicity and Biocompatibility of the SCxPy Hybrid Materials

**Figure 6:**
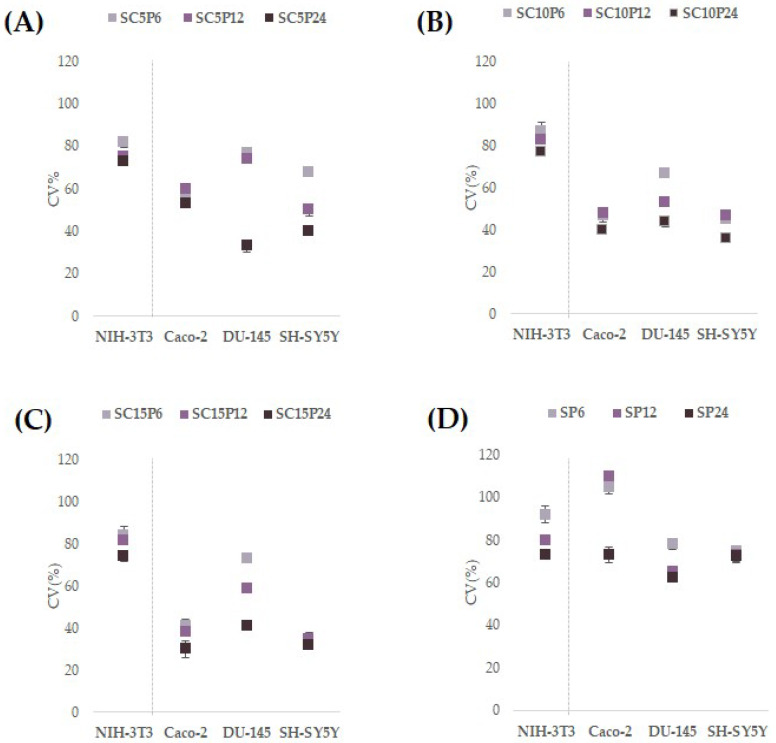
Cell Viability (CV%) of NIH-3T3, Caco-2, DU-145, and SH-SY5Y cell lines after 48 h exposure time with SCP hybrids (**A**–**C**) and SP hybrids (**D**). Values are reported as mean ± SD of three independent measurements carried out on three samples of each synthetized material at 1 mg dose.

To evaluate the biocompatibility of hybrids, an MTT direct test was employed using the mouse fibroblast NIH-3T3 cell line. Furthermore, as chlorogenic acid is antiproliferative towards cancer cells, three different cell lines were selected and tested (Figure 6). The test adopted was always based on the use of 3-(4,5-dimethylthiazol-2-yl)-2,5-diphenyltetrazolium bromide (MTT), which is used as an indicator of overall cytotoxicity [69]. This method is based on the ability of the living cells to reduce dissolved MTT into insoluble formazan in the presence of mitochondrial succinate dehydrogenase [70].

Hybrids, comparable to chlorogenic acid [71], lack toxicity to NIH-3T3, showing its ability to be biocompatible. Furthermore, taking into account that CGA was found to act as antiproliferative towards cancer cells, hybrids were seeded in Petri dishes together with colonic cancer cells, Caco-2, prostate cancer cells, DU-135, and neuroblastoma cells, SH-SY5Y. Both Caco-2 and DU-135 share epithelial morphology. CGA was observed to exert anti-cancer effects on the Caco-2 cancer cell line, leading to the disruption of the distribution of cells in the G0/G1, S, and G2/M phases of the cell cycle [72]. CGA treatment decreased SH-SY5Y cell viability and exhibited drug toxicity at concentrations of 50–400 µM at 12–36 h [73]. All the hybrids showed marked cytotoxic effects against Caco-2 cells, with similar percentage values of reduction of the mitochondrial redox activity in which hybrids with the same concentration of chlorogenic acid but different PCL content are considered. An increased cytotoxicity is observed when, for an equal PCL content, chlorogenic acid is augmented.

The most responsive cells were neuronal ones, which were also previously utilized to evaluate the cytotoxicity of hybrids based on chlorogenic acid and polyethylene glycol [42]. Supporting CGA-induced cytotoxicity, when silica-based hybrids with chlorogenic acid as an organic component were synthesized, it was observed that SH-SY5Y cells changed their phenotype and underwent a reduction in cell viability dependent on the amount of incorporated phenol that the hybrid dose placed directly in contact with them [41]. Considering that PCL in SP hybrids inhibited by a maximum of 25% the cell viability of neuronal cells, while it appeared to increase proliferation of Caco-2 cells, the cytotoxicity detected is likely due to the phenol compound. This is in line with the synthesis of materials in which, while the bioactive molecule loses the ability to transfer hydrogen atoms or single electrons for the exercise of its anti-radical activity, as observed in test-tube tests, it preserves the in vitro effects at the cellular level.

## 3. Materials and Methods

### 3.1. Sol–Gel Synthesis of SCxPy Hybrid Materials

**Figure 7:**
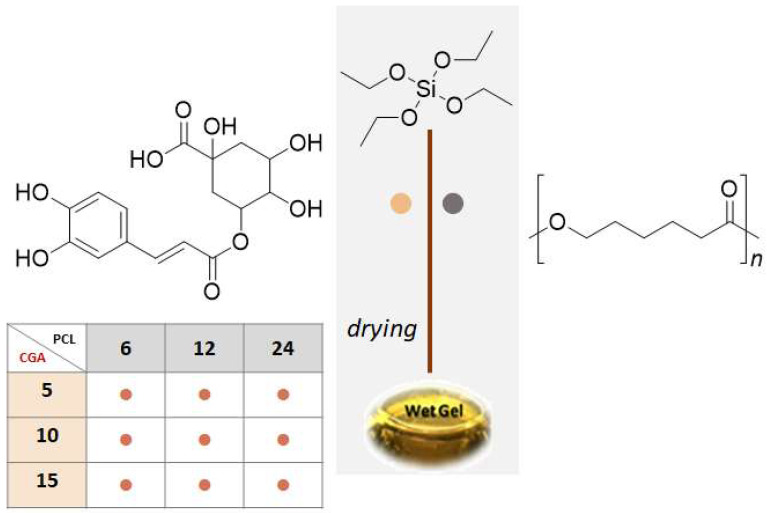
Flowchart of Sol–Gel Synthesis of SCxPy hybrids (●). (●) refers to CGA; (●) refers to PCL.

An inorganic silica solution was created by adding tetraethyl orthosilicate (TEOS, Si(OC_2_H_5_)_4_, Sigma-Aldrich, Darmstadt, Germany) in ethanol (99.8% Sigma-Aldrich). Distilled water and HNO_3_ (≥65%, Sigma-Aldrich) were then added to enhance hydrolysis and condensation kinetics, according to molar ratios equal to TEOS/HNO_3_ = 3.5, EtOH/TEOS = 6.2, and H_2_O/TEOS = 4. Thus, polycaprolactone (PCL; Mw = 14000, Sigma-Aldrich), previously dissolved in chloroform (Sigma-Aldrich), and an ethanolic solution of chlorogenic acid (CGA) was added to the silica solution under continuous stirring. Both PCL and CGA solutions were prepared in order to obtain hybrid materials at 6, 12, or 24 wt% in PCL, and 5, 10, or 15 wt% in CGA. After gelation, synthetized materials were air-dried at 50 °C for 24 h (Figure 7).

### 3.2. Structural Characterization of SCxPy Hybrid Materials

FTIR analyses were performed using a Prestige 21 system (Shimadzu, Japan), with a DTGS KBr (Deuterated Tryglycine Sulphate with Potted Bromide Windows) detector. Sample powders were pressed into KBr, using a Specac manual hydraulic press, to obtain discs (diameter = 12 mm; thickness = 2 mm; weight = 200 mg) with 1 wt% hybrid material content. Transmittance spectra were performed in the 400–4000 cm^−1^ region with a resolution of 4 cm^−1^ (45 scans).

### 3.3. Bioactivity Test

The bioactivity was evaluated following the assay proposed by Kokubo [74,75]. Thus, hybrid material disks were immersed in simulated body fluid (SBF) and maintained at 37 °C for 3 weeks. To avoid the ionic species depletion following biomineral formation, the SBF fluid was restored every two days. FTIR analysis on materials thus treated and properly dried allowed hydroxyapatite to be observed on the surface of the material. The samples were also subjected to scanning electron microscopy (SEM; Quanta 200, FEI, Eindhoven, The Netherlands) to acquire microstructural information on the synthesized hybrid materials energy dispersive X-ray spectroscopy (EDS) analysis for information on the hydroxyapatite layer.

### 3.4. Radical Scavenging Capacity Assessment

The radical scavenging capacity of SCxPy hybrid materials was determined by DPPH and ABTS direct contact tests [42]. For this purpose, a methanolic solution of DPPH (1.0 × 10^−4^ M; 1.0 mL final volume) was poured onto the samples at 1.0 and 2.0 mg, previously placed in a 6 cm Petri dish. After stirring for 30 min at 25 °C, the absorbance at 515 nm was measured against a blank using a PerkinElmer Victor3 multi-label reader (Milan, Italy). Similarly, a solution of ABTS radical cations in PBS (pH 7.4; final volume 1.0 mL) was directly placed in contact with hybrids. The absorbance was measured at 734 nm against a blank, after 6 min of reaction. The results were expressed in terms of the percentage reduction of absorptions of DPPH^●^ or ABTS^●+^ radical by the hybrids.

### 3.5. Biocompatibility Assessment

The direct MTT test was performed on NIH-3T3 fibroblasts, Caco-2 human colorectal adenocarcinoma cells, SH-SY5Y neuroblastoma cells, and DU-145 human prostate cancer cells. Cells were cultured in Dulbecco’s modified Eagle medium supplemented with 10% fetal bovine serum, 50.0 U/mL penicillin, and 100.0 μg/mL streptomycin, at 37 °C in a humidified atmosphere containing 5% CO_2_. Powders of each synthesized material (0.5, 1.0, and 2.0 mg) were placed in 12-well plates, and the cells were seeded (3.5 × 105 cells/well). After 48 h, the medium was removed, and 500 μL of a solution of MTT (3-(4,5-dimethyl-2-thiazolyl)-2,5-diphenyl-2H-tetrazolium bromide; 1.0 mg/mL) added. The treatment was for 2 h at 37 °C in a humidified atmosphere of 5% CO_2_. The MTT solution was then removed, and dimethyl sulfoxide was added for dissolving formazan, whose absorbance was read at 570 nm by the PerkinElmer Victor3 absorbance and fluorescence reader. Cell viability was expressed as the cell mitochondrial redox activity percentage with respect to unexposed cells (100% cell viability; [42]).

### 3.6. Antibacterial Properties of Hybrid Materials

The Kirby–Bauer test was performed using pulverized hybrid materials (100 mg), previously sterilized under UV light for 1 h. *Escherichia coli* (NCTC 13216; Gram-negative), and *Enterococcus faecalis* (ATTC29212; Gram-positive) bacteria were grown with or without hybrid materials [76]. The bacterial suspension of 10^9^ CFU/mL was obtained by diluting the strains in 0.9% sodium chloride solution. *E. coli* was plated on TBX Medium (Tryptone Bile X-Gluc; Liofilchem, Italy), which underwent preliminary sterilization treatment at 120 °C for 15 min. *E. faecalis* was grown on Slanetz Bartley agar medium. *E. coli* plates were incubated at 44 °C for 24 h, while *E. faecalis* plates were incubated at 36 °C for 48 h. The diameter of inhibition halos was calculated as mean (±standard deviation) from three measurements carried out on each sample [77,78].

## 4. Conclusions

Specialized plant metabolites are receiving increasing attention due to their functional versatility. The hypothesis of making a material smart and active through the incorporation/trapping of these compounds in innovative hybrid systems is a valuable strategy. Herein, continuing the sol–gel synthesis of hybrid materials with incorporated small phenolic substances, the synthesis of hybrids made up of an inorganic component based on silica and an organic component with a variable percentage of both PCL and chlorogenic acid has been accomplished. Bioactivity study highlights the hydroxyapatite formation, exploring its functional properties. Moreover, taking into account the plethora of beneficial actions attributed to chlorogenic acid, it has been observed that the hybrids offer promising antibacterial activities. Chlorogenic acid percentage in hybrids seems to play a key role in performing the activity, although PCL content becomes important above all when the polymer is at the highest doses in the sol–gel process. Evaluating the cytotoxicity of the hybrids, the chlorogenic acid functionalization was found to make them biocompatible to the fibroblasts while cytotoxic for the tested cancer lines. These results shed new light on the suitability of synthesized hybrids in the medical field, thus affording knowledge on the features of the bioactive silica–polycaprolactone–chlorogenic acid hybrids.

## Data Availability

The data presented in this study are available on request from the corresponding author.

## Sample Availability

Samples of the compounds are not available from the authors.
